# Nuclear Receptor FTZ-F1 Controls Locust Molt by Regulating the Molting Process of *Locusta migratoria*

**DOI:** 10.3390/insects15040237

**Published:** 2024-03-29

**Authors:** Yichao Zhang, Hongjing Li, Xiaoman Liu, Hongli Li, Qiuyan Lan, Haihua Wu, Yanli Wang, Jianzhen Zhang, Xiaoming Zhao

**Affiliations:** 1 Institute of Applied Biology, Shanxi University, Taiyuan 030006, China; 2 Shanxi Key Laboratory of Nucleic Acid Biopesticides, Taiyuan 030006, China; 3 College of Life Science, Shanxi University, Taiyuan 030006, China

**Keywords:** *Locusta migratoria*, nuclear receptor FTZ-F1, molt, RNA-seq

## Abstract

**Simple Summary:**

Fushi Tarazu Factor-1 (FTZ-F1) is a member of the nuclear receptor superfamily, which plays an important role in the molting process of insects. In this study, two FTZ-F1 transcripts (*LmFTZ-F1-X1* and *LmFTZ-F1-X2*) were identified in *Locusta migratoria*, which were highly expressed in the cuticle. Then, the function of *LmFTZ-F1-X1* and *LmFTZ-F1-X2* in the molting process was explored using RNA interference. Silencing *LmFTZ-F1-X1* and *LmFTZ-F1-X2* separately did not affect the normal development of the nymph, but the simultaneous RNAi of *LmFTZ-F1-X1* and *LmFTZ-F1-X2* blocked the nymphal molting by regulating the genes involved in cuticle formation, chitin synthesis, and other key molting processes.

**Abstract:**

Fushi-tarazu factor 1 (FTZ-F1) is a class of transcription factors belonging to the nuclear receptor superfamily and an important molting regulator in insects; however, its detailed function in the molting process of *Locusta migratoria* is still unclear. This study identified two FTZ-F1 transcripts (*LmFTZ-F1-X1* and *LmFTZ-F1-X2*) in *L. migratoria*. The classical domains of FTZ-F1 were present in their protein sequences and distinguished based on their variable N-terminal domains. Reverse-transcription quantitative polymerase chain reaction analysis revealed that *LmFTZ-F1-X1* and *LmFTZ-F1-X2* were highly expressed in the integument. RNA interference (RNAi) was used to explore the function of *LmFTZ-F1*s in the molting of the third-instar nymph. Separate *LmFTZ-F1-X1* or *LmFTZ-F1-X2* silencing did not affect the normal development of third-instar nymphs; however, the simultaneous RNAi of *LmFTZ-F1-X1* and *LmFTZ-F1-X2* caused the nymphs to be trapped in the third instar stage and finally die. Furthermore, the hematoxylin-eosin and chitin staining of the cuticle showed that the new cuticles were thickened after silencing the *LmFTZ-F1*s compared to the controls. RNA-seq analysis showed that genes encoding four cuticle proteins, two chitin synthesis enzymes, and cytochrome P450 303a1 were differentially expressed between ds*GFP*- and ds*LmFTZ-F1*s-injected groups. Taken together, *LmFTZ-F1-X1* and *LmFTZ-F1-X2* are involved in the ecdysis of locusts, possibly by regulating the expression of genes involved in cuticle formation, chitin synthesis, and other key molting processes.

## 1. Introduction

Integuments play multiple roles in the growth and development of insects and help avoid pesticide damage and water loss [1,2]. Moreover, metamorphosis is a crucial characteristic during insect growth and development, and ecdysis is the driving force for metamorphosis, which consists of old integument degradation and new integument generation [3]. The main components of insect cuticles are chitin, proteins, and lipids, the quantity and structure of which experience great changes in the molting process of insects, such as chitin degradation, chitin synthesis, and chitin–protein interactions [4,5,6]. Many genes are involved in these processes. For example, chitinase and chitin synthesis genes are involved in chitin degradation and synthesis during molting [7], cuticle protein genes are essential for the formation of the cuticle structure [6], and the cytochrome p450 4G family can catalyze the synthesis of cuticular hydrocarbons [8]. Moreover, the disruption of the expression of these genes leads to abnormal molting; for example, the RNAi-mediated inhibition of chitinase and chitin synthesis enzyme genes leads to severe molting defects and lethality in *Leptinotarsa decemlineata*, *Tribolium castaneum*, and *Locusta migratoria* [9,10,11,12], while the RNAi of the cuticle protein *TcCP30* prevented eclosion in *T. castaneum* [13]. These key molting genes are regulated by many transcription factors involved in the ecdysteroid signaling pathway, such as nuclear receptor (NR) genes, which are an important class of transcription factors involved in insect metamorphosis [14]. The function of several NR genes has been clarified in *L. migratoria*, such as nuclear receptors *LmHR3* and *LmHR4*, which control metamorphosis by regulating chitin synthesis and degradation genes, and nuclear receptor LmHR39, which controls molting by regulating carboxypeptidase and chitinase genes, which are involved in old cuticle degradation during metamorphosis [15,16,17].

In addition to *HR3*, *HR4*, and *HR39* genes, Fushi-tarazu factor 1 (FTZ-F1) is an important member of the nuclear receptor superfamily, which is belong to the NR5 family [18]. To date, *FTZ-F1* genes have been identified in some insects, and only one *FTZ-F1* transcript has been cloned in most insects, including *Helicoverpa armigera*, *Plutella xylostella*, and *Blattella germanica* [19,20,21]. However, there were two *FTZ-F1* gene isoforms in several insects; for example, *αFTZ-F1* and *βFTZ-F1* were cloned in *Drosophila melanogaster* [22]. The function of FTZ-F1 has been clarified in several insects, and it is a response factor for 20-hydroxyecdysone (20E) and juvenile hormone (JH). In *Manduca sexta* and *D. melanogaster*, the expression level of *FTZ-F1* was up-regulated after a 20E titer decline [23], whereas JH III induced the appearance of the FTZ-F1 protein in *Aedes aegypti* [24]. Moreover, *FTZ-F1* plays an important role in metamorphosis, and RNAi-mediated FTZ-F1 depletion and mutated *βFTZ-F1* induced the defective eclosion in *D. melanogaster* [25]; in *H. armigera,* silencing *HaFTZ-F1* blocks nymphal molting in the fourth instar [19]; and in *Lasioderma serricorne*, the RNAi of *LsFTZ-F1* prevented nymphal pupal molting [26]. Although FTZ-F1 is involved in the molting process of some insects, its detailed function in the ecdysis of *L. migratoria* remains unclear.

*L. migratoria* is a widespread pest of grains worldwide, which undergoes six developmental stages, with the first five being nymphal stages and the final stage being the adult stage [27]. Genes involved in ecdysis are potential targets for pest management [1,2], and *FTZ-F1* is an important regulator of ecdysis in insects [25]; therefore, what is the specific function of *FTZ-F1* in the molting process of *L. migratoria*? In this study, *LmFTZ-F1* genes were identified from the transcriptomic databases of *L. migratoria*. RNA interference (RNAi) technology was used to explore the functions of *LmFTZ-F1* during molting. Furthermore, potential downstream targets of *LmFTZ-F1* were screened using RNA-seq. These results lay the foundation for screening green molecular targets for *L. migratoria* control.

## 2. Materials and Methods

### 2.1. Insect Rearing

The eggs of *L. migratoria* were purchased from Insect Protein Co., Ltd. (Cangzhou, China). Nymphs were reared in a rearing cage at 30 ± 2 °C with 40 ± 5% relative humidity under a 14 h:10 h light/dark cycle. The nymphs were then fed wheat seedlings.

### 2.2. Gene Identification of LmFTZ-F1

The cDNA sequence of *LmFTZ-F1* was obtained from a *L. migratoria* transcriptome database [15]. The exon/intron prediction of the *LmFTZ-F1* gene was performed by comparing the coding sequence of *LmFTZ-F1* with the locust genome sequence using the NCBI BLAST tool [27]. The prediction of open reading frames (ORFs) and translation of the cDNA sequences into amino acid sequences were performed using the translation tool ExPaSy (http://web.expasy.org/translate/, accessed on 1 October 2020). The SMART tool (http://smart.embl.de/, accessed on 26 May 1998) was used to predict conserved protein motifs. Five FTZ-F1 protein sequences from different insects were chosen to perform amino acid sequence alignments using GeneDoc version 2.7.0 (Free software foundation, Inc., Boston, MA, USA) [28]. A phylogenetic tree was constructed using MEGA software (version 5.0) with the neighbor-joining method using 1000 repetitions [29].

### 2.3. Tissue- and Stage-Dependent Expression Analysis of LmFTZ-F1

To analyze the tissue-dependent expression of *LmFTZ-F1*, six tissues, including the gastric cecum, fat body, foregut, hindgut, midgut, and integument, were dissected on day 5 of the third-instar nymphs (N3D5). In addition, to analyze the stage-dependent expression of *LmFTZ-F1*, third-instar nymphs (N3D1-N3D5) were collected. Total RNAs were extracted from the whole body and different tissues using RNAiso Plus (TaKaRa, Tokyo, Japan) according to the manufacturer’s instructions. The concentration of total RNAs was determined using a NanoDrop 2000 spectrophotometer (Thermo, Inc., Waltham, MA, USA). The All-In-One 5× RT MasterMix (+gDNA wiper) Kit (ABM, Nanjing, China) was used to synthesize first- strand cDNA. Briefly, 1 μg of total RNA dissolved in 16 μL RNase-free water and 4 μL All-In-One 5× RT MasterMix were added to the reaction system; then, the reaction was conducted at 37 °C for 15 min, 60 °C for 10 min, and 95 °C for 3 min to synthesize the cDNA.

Reverse-transcription quantitative polymerase chain reaction (RT-qPCR) was performed using MonAmp™ SYBR^®^ Green qPCR Mix (None/Low/High ROX) (Monad Biotech Co., Ltd., Wuhan, China) and a LightCycler^®^ 480 Real-Time PCR System (Roche Diagnostics GmbH, Mannheim, Germany). The reaction system contained 10 μL MonAmp™ SYBR^®^ Green qPCR Mix, 1 μL of forward and reverse primer (10 μM), 3 μL of 5-fold diluted cDNA, and 6 μL ddH_2_O. The temperature procedure consisted of the first step at 95 °C for 5 min, followed by 40 cycles of 95 °C for 15 s and 60 °C for 31 s. Melting curves were analyzed for each primer. The transcription level of each gene was calculated using the 2^−ΔΔCt^ method [30]. *LmEF-1α* and *LmGAPDH* were the reference genes for calibrating the expression level of the target genes [31]. All samples were prepared as three independent biological replicates, with each replicate containing three nymphs. The primers used in the experiments are listed in Table S2.

### 2.4. Functional Analysis of LmFTZ-F1 by RNAi

In order to study the biological functions of *LmFTZ-F1*, double-stranded RNA (dsRNA) of green fluorescent proteins (ds*GFP*), *LmFTZ-F1-X1* (ds*LmFTZ-F1-X1*), *LmFTZ-F1-X2* (ds*LmFTZ-F1-X2*), and two isoforms of the *LmFTZ-F1* gene (ds*LmFTZ-F1*s) were synthesized using T7 RiboMAX™ Express RNAi System (Promega, Inc. Madison, WI, USA) as previous report [15]. The PCR products of specific sequences of *LmFTZ-F1-X1* and *LmFTZ-F1-X2* were selected as templates for synthesizing ds*LmFTZ-F1-X1* and ds*LmFTZ-F1-X2*, respectively. Ds*LmFTZ-F1-X1* and ds*LmFTZ-F1-X2* were used to inhibit the expression of *LmFTZ-F1-X1* and *LmFTZ-F1-X2*, respectively. The PCR products of the common sequences from *LmFTZ-F1-X1* and *LmFTZ-F1-X2* were used to synthesize efficient ds*LmFTZ-F1*s to inhibit the expression of *LmFTZ-F1-X1* and *LmFTZ-F1-X2* together. Ten micrograms of different dsRNAs were injected into the abdomen on day 1 of the third instar (N3D1). A Ds*GFP*-injected group was used as the control. Each treatment contained three independent biological replicates, and eight nymphs were included in each replicate. After dsRNA injection, the nymphs were reared normally for phenotypic investigation. At 24 h after dsRNA injection, the integuments of dsRNA-injected nymphs were dissected to analyze the silencing efficiency using RT-qPCR. The samples were prepared as three independent biological replicates, with each replicate containing three nymphs. The primers used in the experiments are listed in Table S2.

### 2.5. Microsection and Hematoxylin-Eosin (H&E) Staining of the Cuticle

To further study the effects of *LmFTZ-F1* RNAi on the molting process, H&E staining of the cuticle was performed as previously described [32]. Briefly, nymphs were injected with 10 μg of ds*GFP* and ds*LmFTZ-F1*s on day 1 of the third instar, and the second abdominal cuticles from ds*GFP*- and ds*LmFTZ-F1*s-injected nymphs were dissected on day 5 of the third instar (N3D5). Then, the dissected cuticles were fixed and used for making paraffin section (5 μm), and finally stained with hematoxylin-eosin. The stained paraffin sections were observed under an Olympus BX51 microscope (Olympus, Tokyo, Japan) and photographed using an Olympus digital camera.

### 2.6. Microsection and Chitin Staining

To study the impacts of *LmFTZ-F1*s RNAi on chitin formation, microsections and chitin staining of cuticle were conducted as previously reported [33]. Briefly, paraffin sections (5 μm) of the second abdominal cuticle from ds*GFP*- and ds*LmFTZ-F1*s-injected nymphs were prepared on day 5 of the third instar. Then, the chitin was stained with Fluorescent Brightener 28 (FB28) (Sigma, Inc. St Louis, MO, USA) (1 mg/mL), and the nuclei were labeled with SYTOX™ Green Nucleic Acid Stain (Thermo Fisher Scientific, Waltham, MA, USA) (25 μg/mL) [34]. An LSM 880 confocal laser scanning microscope (Zeiss, Inc., Oberkochen, Germany) was used to capture images of the stained samples.

### 2.7. RNA-Seq Analysis

The total RNA of the integuments from ds*GFP*- and ds*FTZ-F1*s-injected nymphs was extracted on day 5 from the third-instar nymphs (N3D5). A cDNA library was constructed and sequenced on an Illumina NovaSeq6000 platform (Illumina, San Diego, CA, USA) in Biomarker Technologies (Beijing, China). After obtaining the raw sequence data, reads that included adapters and low-quality reads (Q < 10) were removed from the raw data. The clean reads were aligned with the locust genome using HISAT version 3.0 (Hopkins, Baltimore, MD, USA) [27,35], and the StringTie program was used to assemble the short reads [36]. For functional annotation, the assembled unigenes were compared with the COG, NR, KOG, Swiss-Prot, and KEGG databases using the DIAMOND version 0.9.29 (Tuebingen U, Tübingen, Germany) [37], and GO annotation and classification for unigenes were performed using InterProScan version 5.0 (EMBL-EBI, Cambridge, UK) [38]. For the annotation of newly predicted genes, the protein sequences of the newly predicted genes were aligned with the pfam database using HMMER version 3.0 (WUSM, St Louis, MO, USA) [39]. Based on the counts of mapped reads, gene expression levels were normalized using fragments per kilobase of transcript per million fragments mapped values (FPKM) [40]. DESeq2 version 1.10.1 (EMBL, Heidelberg, Germany) was used to identify differentially expressed genes (DEGs) [41]. The parameters were a false discovery rate (FDR) ≤ 0.05 and |log2FC| (FC, fold change) ≥ 1.5. RT-qPCR was used to verify the differentially expressed genes identified through RNA-seq and detect the silencing efficiency on day 5 of the third-instar nymphs injected with ds*LmFTZ-F1*s, as described above. The integuments of ds*GFP*-, ds*LmFTZ-F1*-*X1-*, and ds*LmFTZ-F1*-*X2*-injected nymphs were dissected on day 5 of the third instar to assess the efficacy of gene silencing and determine the expression levels of DEGs identified through RNA-seq analysis.

### 2.8. Data Analysis

Statistical analyses were performed using the SPSS software (version 19.0; SPSS Inc., Chicago, IL, USA). Significant differences in expression among different developmental stages and tissues were analyzed using Tukey’s HSD multiple comparison test. Other data analyses were performed using independent-samples *T*-tests.

## 3. Results

### 3.1. Bioinformatic Analysis of LmFTZ-F1

**Figure 1:**
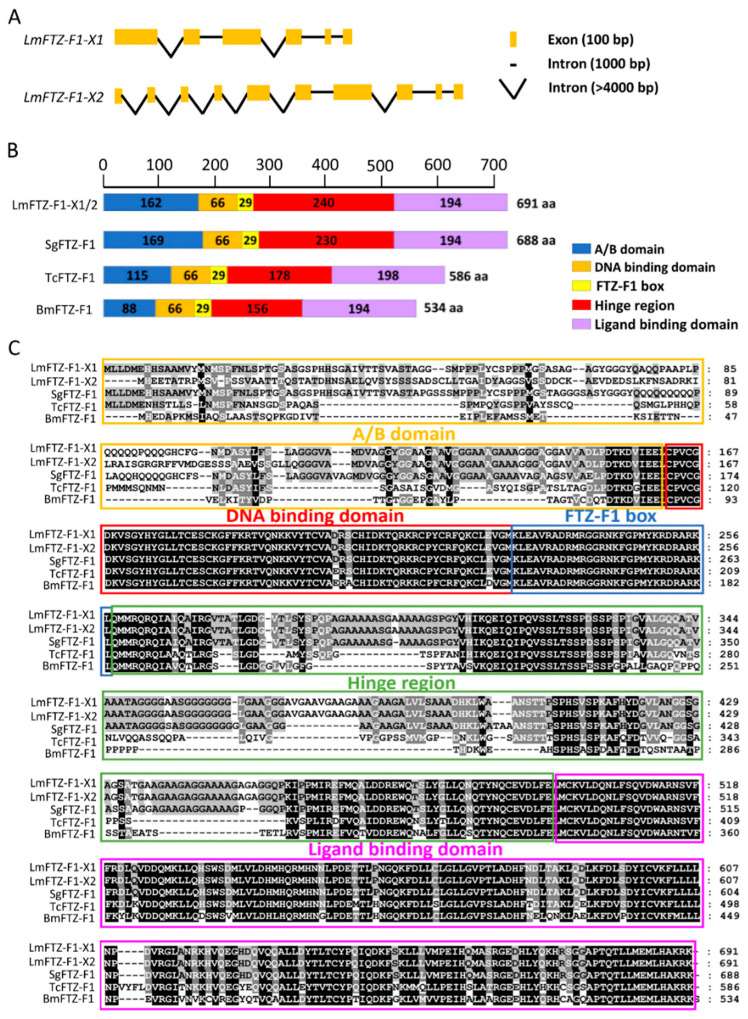
Gene structure of *LmFTZ-F1*s in *L. migratoria*. (**A**) Genomic structure of *LmFTZ-F1-X1* and *LmFTZ-F1-X2*. Orange boxes represent exons and black lines represent introns. (**B**) Conserved motif comparison of LmFTZ-F1s with other FTZ-F1s from different insects. The numbers in domains represent the number of amino acids in this domain, and each domain is represented by different colors. Sequences were from *L. migratoria* (Lm), *Schistocerca gregaria* (Sg), *Tribolium castaneum* (Tc), and *Bombyx mori* (Bm). aa: amino acid. (**C**) Multiple sequence alignments of the deduced FTZ-F1 proteins in different insects. The N-terminal A/B domain, DNA binding domain, FTZ-F1 box, hinge region, and ligand binding domain were enclosed in golden, red, blue, green and purple boxes, respectively. The sequences on the black background are completely conservative, while the sequences on the gray background show partial conservatism.

**Figure 2:**
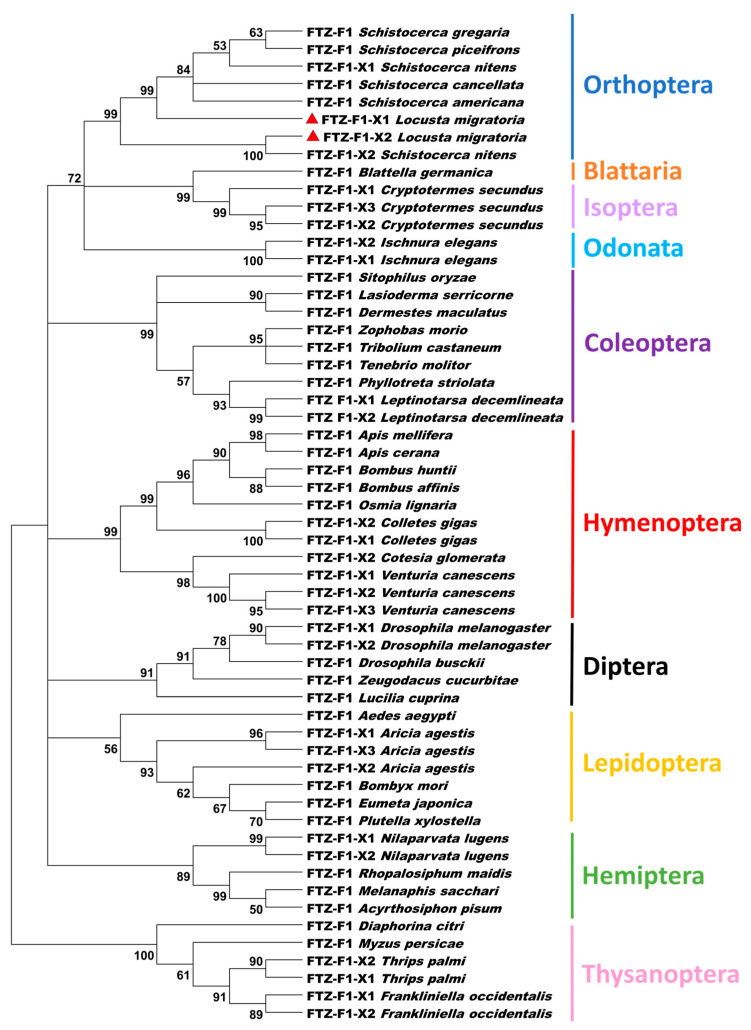
Phylogenic analysis of the FTZ-F1s from different insects. The phylogenetic tree was constructed using MEGA 5.0. LmFTZ-F1-X1 and LmFTZ-F1-X2 are marked with red triangles. The GenBank accession numbers of FTZ-F1 sequences are listed in Table S1.

Two isoforms of the *LmFTZ-F1* gene were identified in the locust transcriptome, which were named as *LmFTZ-F1-X1* and *LmFTZ-F1-X2*. The *LmFTZ-F1-X1* and *LmFTZ-F1-X2* genes included six and ten exons, respectively; the last five exons of *LmFTZ-F1-X1* and *LmFTZ-F1-X2* were identical, and alternative splicing occurred in exon1 of *LmFTZ-F1-X1* and exon1-4 of *LmFTZ-F1-X2* (Figure 1A). *LmFTZ-F1-X1* and *LmFTZ-F1-X2* contained the coding sequences of 2076 bp encoding 691 amino acids. The classical domains (a variable N-terminal domain, DNA-binding domain, FTZ-F1 box, ligand-binding domain, and hinge region) of FTZ-F1 were present in the LmFTZ-F1-X1 and LmFTZ-F1-X2 protein sequences (Figure 1B,C), and LmFTZ-F1-X1 and LmFTZ-F1-X2 were distinguished based on the variable N-terminal domain (A/B domain) (Figure 1C). LmFTZ-F1 and 56 FTZ-F1 protein sequences from different insects were collected for phylogenetic analysis, as shown in Figure 2. LmFTZ-F1-X1 and LmFTZ-F1-X2 were clustered with the FTZ-F1s of *Schistocerca*, all of which constituted the Orthoptera branch close to the Blattaria and Isoptera branches (Figure 2).

### 3.2. Tissue and Developmental Expression Patterns of LmFTZ-F1

**Figure 3:**
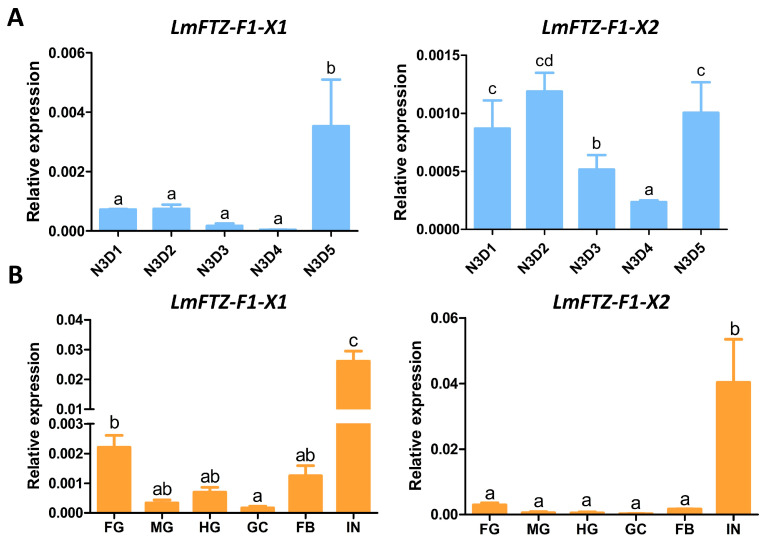
Expression analysis of *LmFTZ-F1-X1* and *LmFTZ-F1-X2* in different stages and tissues. (**A**) The expression patterns of *LmFTZ-F1-X1* and *LmFTZ-F1-X2* in third-instar nymphs. N3D1-N3D5: day 1 to day 5 of third instar. (**B**) The expression patterns of *LmFTZ-F1-X1* and *LmFTZ-F1-X2* in different tissues. FG: foregut; MG: midgut; HG: hindgut; GC: gastric caecum; FB: fat body; IN: integument. The data were analyzed using Tukey’s HSD multiple comparison test. The different letters above the bar represent significant differences among these samples (*p* < 0.05).

The expression levels of *LmFTZ-F1-X1* and *LmFTZ-F1-X2* in third-instar nymphs were analyzed using RT-qPCR. As shown in Figure 3, during the third instar, *LmFTZ-F1-X1* was expressed at a low level from day 1 to day 4 and increased immediately on day 5. The transcription level of *LmFTZ-F1-X2* on the early (day 1–2) and final (day 5) days was relatively higher than that on middle days (day 3–4) (Figure 3A). Moreover, we explored the expression patterns of *LmFTZ-F1-X1* and *LmFTZ-F1-X2* in different tissues of N3D5 nymphs, and found that *LmFTZ-F1-X1* and *LmFTZ-F1-X2* were highly expressed in the integuments (Figure 3B).

### 3.3. Effect on Nymphal Survival after LmFTZ-F1 RNAi

**Figure 4:**
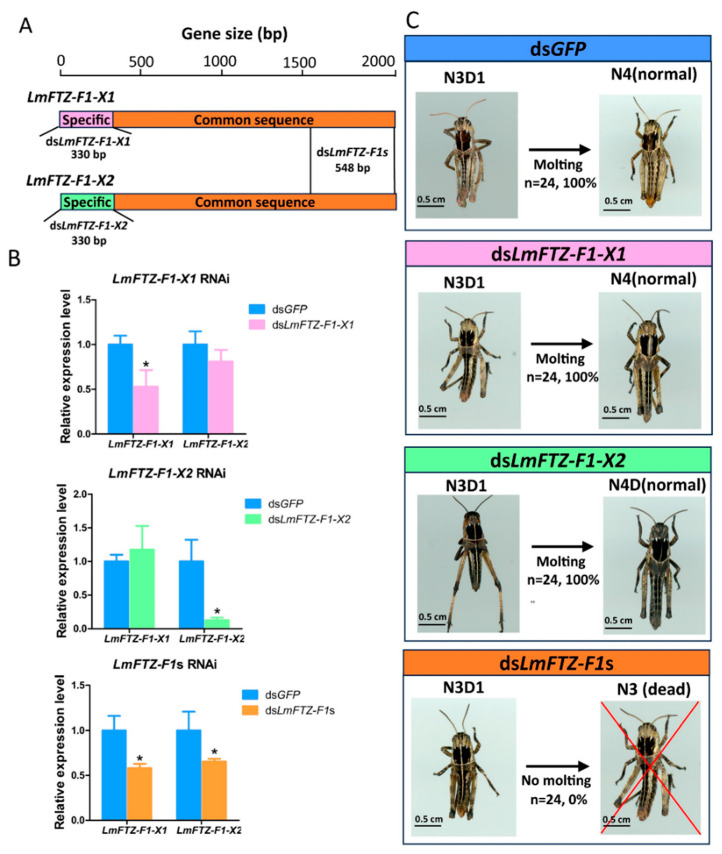
Effects of separate and simultaneous *LmFTZ-F1-X1* and *LmFTZ-F1-X2* silencing on the nymphal–nymphal molting in *L. migratoria*. (**A**) The dsRNA template for separate and simultaneous *LmFTZ-F1-X1* and *LmFTZ-F1-X2* silencing. The specific sequences of *LmFTZ-F1-X1* and *LmFTZ-F1-X2* were selected to synthesize ds*LmFTZ-F1-X1* and ds*LmFTZ-F1-X2*, respectively. The common sequence of *LmFTZ-F1-X1* and *LmFTZ-F1-X2* was selected to synthesize ds*LmFTZ-F1*s. (**B**) Expression analysis of *LmFTZ-F1-X1* and *LmFTZ-F1-X2* at 24 h after injecting ds*GFP*, ds*LmFTZ-F1-X1*, ds*LmFTZ-F1-X2*, and ds*LmFTZ-F1*s. The data were analyzed using the independent-samples *T*-test. The asterisks indicated that there were significant differences between the control and treatment groups (* *p* < 0.05). (**C**) Phenotypic analysis of separate and simultaneous *LmFTZ-F1-X1* and *LmFTZ-F1-X2* silencing; the percentage represents the proportion of normal molting from the third to the fourth instar. N3D1: day 1 of the third instar; N3: third instar; N4: fourth instar.

To investigate the biological function of *LmFTZ-F1* in the ecdysis of *L. migratoria*, specific sequences of *LmFTZ-F1-X1* and *LmFTZ-F1-X2* were selected to synthesize ds*LmFTZ-F1-X1* and ds*LmFTZ-F1-X2*, respectively (Figure 4A), and Ds*GFP*, ds*LmFTZ-F1-X1*. and ds*LmFTZ-F1-X2* were injected into the locusts on day 1 of the third instar, respectively. 1. After 24 hours of the separate injections with ds*LmFTZ-F1-X1* and ds*LmFTZ-F1-X2*, the expression levels of *LmFTZ-F1-X1* and *LmFTZ-F1-X2* were significantly reduced to 0.53-fold and 0.13-fold of the control, respectively (Figure 4B). Resembling the ds*GFP*-injected nymphs, the nymphs separately injected with ds*FTZ-F1-X1* and ds*FTZ-F1-X2* successfully molted into fourth-instar nymphs on day 6 of the third instar (Figure 4C). Moreover, a common sequence between *LmFTZ-F1-X1* and *LmFTZ-F1-X2* was selected to synthesize the ds*LmFTZ-F1*s (Figure 4A). At 24 h after ds*LmFTZ-F1*s injection, the expression of *LmFTZ-F1-X1* and *LmFTZ-F1-X2* was reduced to 0.58- and 0.65-fold of the control, respectively (Figure 4B). The ds*LmFTZ-F1*s-injected third-instar nymphs failed to develop into fourth-instar nymphs and finally died in the third instar (Figure 4C), indicating that *LmFTZ-F1-X1* and *LmFTZ-F1-X2* were involved in nymphal–nymphal molting.

### 3.4. Effects of LmFTZ-F1s RNAi on Cuticle Formation of L. migratoria

**Figure 5:**
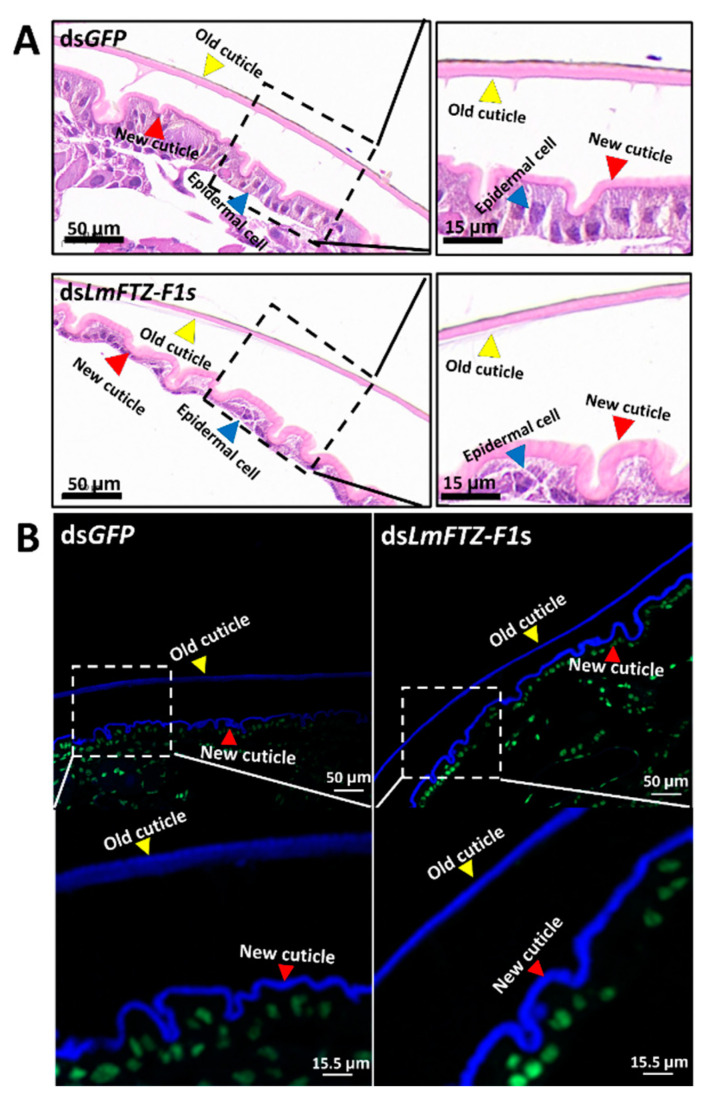
Analysis of the microscopic structure of the integument after *LmFTZ-F1-X1* and *LmFTZ-F1-X2* depletion. (**A**) Microsection and hematoxylin-eosin (H&E) staining of the nymphal cuticle on day 5 of the third instar after the simultaneous *LmFTZ-F1-X1* and *LmFTZ-F1-X2* silencing. The red, yellow, and blue triangles represent new cuticle, old cuticles, and epidermal cells, respectively. (**B**) Chitin staining of the cuticle of nymphs on day 5 of the third instar after the simultaneous *LmFTZ-F1-X1* and *LmFTZ-F1-X2* silencing. The nuclei (green) were labeled with 25 μg/mL SYTOX™ Green Nucleic Acid Stain (Thermo Fisher Scientific, Waltham, MA, USA), and the chitin (blue) was stained with Fluorochrome28.

To explore how *LmFTZ-F1*s RNAi affected the molting process of *L. migratoria*, integument microsections of ds*GFP*- and ds*LmFTZ-F1*s-injected N3D5 nymphs were prepared for H&E and chitin staining. The results revealed that the new cuticles of the ds*GFP*-injected nymphs were thinner than those of the ds*LmFTZ-F1*s-injected group (Figure 5A,B). Moreover, the epidermal cell arrangement was disrupted after the simultaneous RNAi of *LmFTZ-F1-X1* and *LmFTZ-F1-X2* (Figure 5A).

### 3.5. Differentially Expressed Genes after LmFTZ-F1s RNAi

**Figure 6:**
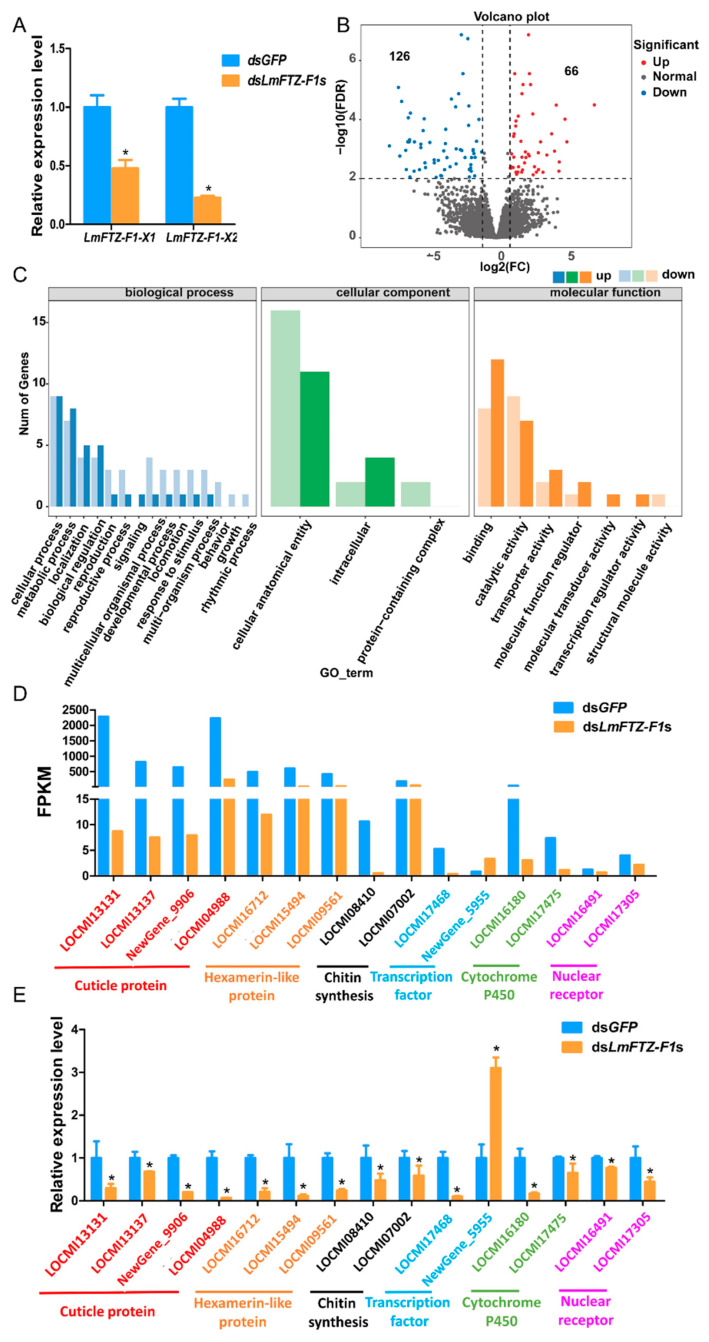
RNA sequencing analysis after the simultaneous *LmFTZ-F1-X1* and *LmFTZ-F1-X2* silencing. (**A**) Expression analysis of *LmFTZ-F1-X1* and *LmFTZ-F1-X2* on day 5 of the third-instar nymphs injected with ds*GFP* and ds*LmFTZ-F1*s. The data were analyzed using the independent-samples *T*-test. The asterisks indicated that there were significant differences between the control and treatment groups (* *p* < 0.05). (**B**) MA plot of differentially expressed genes between the ds*GFP*- and ds*LmFTZ-F1*s-injected groups. FC, fold change. (**C**) The classification of differentially expressed genes in biological processes, cellular components, and molecular function. (**D**) Differentially expressed genes encoding cuticle proteins, hexamerin-like proteins, chitin synthesis enzymes, transcription factors, cytochrome P450 enzymes, and nuclear receptors in the transcriptome data. FPKM: fragments per kilobase of exon per million fragments mapped. (**E**) The relative expression of differentially expressed genes between ds*GFP*- and ds*LmFTZ-F1*s-injected nymphs were analyzed using RT-qPCR on day 5 of the third instar. The data were analyzed using the independent-samples *T*-test. The asterisks indicated that there were significant differences between the control and treatment groups (* *p* < 0.05).

To explore why *LmFTZ-F1*s RNAi led to death before molting, an RNA-seq analysis of the integument from ds*GFP-* and ds*LmFTZ-F1*s-injected nymphs was conducted on day 5 of the third instar. After injecting ds*LmFTZ-F1*s on day 1 of the third instar, the expression level of *LmFTZ-F1-X1* and *LmFTZ-F1-X2* was reduced 0.53- and 0.78-fold, respectively, compared to that in the controls on day 5 of third instar (Figure 6A). A total of 22,005 genes were identified, and 192 genes were differentially expressed between the ds*GFP*- and ds*LmFTZ-F1*s-injected groups, including 66 up-regulated genes and 126 down-regulated genes in the ds*LmFTZ-F1*s-injected group compared with the control (Figure 6B). According to the Gene Ontology classification of the differentially expressed genes, in the biological process category, cellular and metabolic processes were the major subcategories, the cellular anatomical entity was the largest subcategory in the cellular component category, and the molecular function category was dominated by the binding and catalytic activity subcategories (Figure 6C). According to the annotation of DEGs (Table S3), the genes encoding four cuticle proteins (nymph cuticular protein NCP62, nymph cuticular protein NCP9.5, cuticle protein 16.5-like, and cuticle protein 6.4), three hexamerin-like proteins (two hexamerin-like protein 1s and hexamerin-like protein 2), two chitin synthesis enzymes (trehalase and glutamine--fructose-6-phosphate aminotransferase), two transcription factors (BTB/POZ domain-containing protein 9 and metabotropic glutamate receptor 3), two cytochrome P450 enzymes (cytochrome P450 303a1 and cytochrome P450 4C1-like), and two nuclear receptors (hormone receptor 3 and hormone receptor 4 isoform X2) were differentially expressed between the ds*GFP*- and ds*LmFTZ-F1*s-injected groups (Figure 6D,E). These genes may be involved in the molting process. In addition, the expression levels of DEGs induced by the co-silencing of *LmFTZ-F1-X1* and *LmFTZ-F1-X2* were detected after silencing *LmFTZ-F1-X1* and *LmFTZ-F1-X2* separately, and the expression levels of most DEGs remained unchanged after silencing *LmFTZ-F1-X1* and *LmFTZ-F1-X2* separately (Figure S1C,D).

## 4. Discussion

Insect molting includes a cascade of imperceptible changes driven by many key molting genes, such as chitin synthesis and degradation genes [7]. Nuclear receptors are an important class of transcription factors involved in insect metamorphosis that regulate the expression of key molting genes [14]. As a member of the nuclear receptor superfamily, FTZ-F1 is involved in the molting process of many insects [19,25,26]; however, its detailed function in the molting process of *L. migratoria* remains unclear.

In the present study, we found two isoforms of FTZ-F1 (*LmFTZ-F1-X1* and *LmFTZ-F1-X2*) in *L. migratoria*, which were analogous to those in *D. melanogaster* and *L. decemlineata* [22,42]. The sequence alignment of LmFTZ-F1 showed that the conserved DNA- and ligand-binding domains of nuclear receptor were present in their protein sequences, which act as an important bridge between the hormone-response genes and corresponding hormones [14]. The FTZ box of LmFTZ-F1 was completely consistent with that of other insects, which was used to enhance the DNA-binding specificity [18]. Resembling other insects, the distinguishing symbols of LmFTZ-F1-X1 and LmFTZ-F1-X2 are located in the variable N-terminal domain (A/B domain) [22,42]. In addition, a phylogenetic analysis, constructed using LmFTZ-F1 and 56 other FTZ-F1s, showed that LmFTZ-F1-X2 was close to the SnFTZ-FX2 of *Schistocerca nitens*, whereas LmFTZ-F1-X1, SnFTZ-FX1, and other LmFTZ-F1s of *Schistocerca* were clustered into one group in the Orthoptera branch, which further indicated that there were some differences in the function of LmFTZ-F1-X1 and LmFTZ-F1-X2. Moreover, the FTZ-F1s of the Orthoptera branch were closely related to the FTZ-F1s of Isoptera and Blattaria, confirming that the FTZ-F1s were conserved in hemimetabolous insects. The current literature predominantly focuses on the role of FTZ-F1 in insect molting within holometabolous insects, such as *P. xylostella*, *D. melanogaster*, and *H. armigera* [19,20,22]. In hemimetabolous insects, only one isoform of *FTZ-F1* has been reported in *Blattella germanica*, and it has only been shown to be functional in the transition from nymph to adult [21]. In this research, two isoforms of *FTZ-F1* were identified in *L. migratoria*, which serves as a valuable model for investigating the function of *FTZ-F1* in hemimetabolous insects.

To explore the function of *LmFTZ-F1*, we first detected the expression of *LmFTZ-F1-X1* and *LmFTZ-F1-X2* in third-instar nymphs. During the third instar, *LmFTZ-F1-X1* and *LmFTZ-F1-X2* were both highly expressed on the last day of the third instar; moreover, the expression of *LmFTZ-F1-X2* was high in the initial stage of the third instar. The developmental expression patterns of *LmFTZ-F1*s were analogous to those in other insects. For example, the transcript level of *LdFTZ-F1* is high after molting and decreases significantly during the middle instar stages of *L. decemlineata* [42]. The expression of *HaFTZ-F1* increased sharply before or immediately after each molting in the larval stage of *H. armigera* [19]. Moreover, in *L. migratoria*, the nuclear receptor *LmHR4*, a key gene involved in cuticle formation, was expressed at its highest level on the last day of each instar [16]. In addition, *LmFTZ-F1-X1* and *LmFTZ-F1-X2* were highly expressed in the integument, which was analogous to other insects. For example, in *H. armigera* and *Henosepilachna vigintioctopunctata*, the *FTZ-F1*s are highly expressed in the epidermis [19,43]. The high expression of *LmFTZ-F1* in the integument before molting indicated that *LmFTZ-F1* might be involved in cuticle formation during molting.

RNAi technology was used to study the function of *LmFTZ-F1*. The separate knockdown of *LmFTZ-F1-X1* and *LmFTZ-F1-X2* did not affect the normal development of the third-instar nymphs. However, simultaneous *LmFTZ-F1-X1* and *LmFTZ-F1-X2* silencing caused nymphs to be arrested in the third instar and finally die. Similarly, in other insects, the RNAi of *FTZ-F1* blocks normal development in the nymphal phase. *BgFTZ-F1* depletion leads to the failure of the larval–larval molting in *B. germanica* [21]. The RNAi of *HaFTZ-F1* in the fourth-instar nymph blocked nymphal ecdysis in *H. armigera* [19]. Like *L. migratoria*, two *FTZ-F1* isoforms (*HvαFTZ-F1* and *HvβFTZ-F1*) were identified in *H. vigintioctopunctata*, and the third-instar nymphs separately injected with ds*HvαFTZ-F1* or ds*HvβFTZ-F1* successfully developed into the fourth instar. However, simultaneous *HvαFTZ-F1* and *HvβFTZ-F1* silencing prevented the molting from the third to the fourth instar [43], consistent with our results. In addition, the expression levels of DEGs induced by the co-silencing of *LmFTZ-F1-X1* and *LmFTZ-F1-X2* were detected after silencing *LmFTZ-F1-X1* and *LmFTZ-F1-X2* separately, and the expression level of most DEGs remained unchanged after silencing *LmFTZ-F1-X1* and *LmFTZ-F1-X2* separately, which indicated that silencing *LmFTZ-F1-X1* and *LmFTZ-F1-X2* separately might not affect the expression of genes critical for successful molting. In *Lepeophtheirus salmonis*, silencing *αFTZ-F1* neither caused apparent phenotypes in the larvae and adults, nor changed the expression of other related genes, as determined by RNA sequencing and qRT-PCR [44]. All of these indicated that there may be some functional redundancy between *LmFTZ-F1-X1* and *LmFTZ-F1-X2*.

To understand how *LmFTZ-F1*s depletion specifically affects nymphal–nymphal molting, we analyzed the structural changes in the integument after silencing *LmFTZ-F1*s. We found that the new cuticles of ds*LmFTZ-F1*s-injected nymphs were thicker than those of the ds*GFP*-injected group. As an important component of insect cuticle, CPR proteins form horizontal sheets (laminae) by interacting with chitin. These laminae are stacked helicoidally or with unidirectional microfibril orientation along the vertical axis of the cuticle, and the cuticle thickness depends not only on the number of the laminae, but also on their arrangement [45,46]. And the silencing of CPR genes would destroy lamellar arrangement; for example, in *T. castaneum*, the RNAi of the cuticle protein *TcCPR27* caused the laminae to arrange loosely, resulting in a thickening of the cuticle [46]. In our study, cuticle protein genes were significantly down-regulated in the ds*LmFTZ-F1*s-injected group compared to the control group according to the RNA-seq and RT-qPCR analysis (Table S3, Figure 6D,E), indicating that the silencing of *LmFTZ-F1*s might impair the cuticle structure of locusts by regulating the expression of cuticle protein genes. Moreover, in our research, the epidermal cell arrangement was disrupted after silencing *LmFTZ-F1*s. During the molting process, epidermal cells are activated by ecdysone and subsequently undergo proliferation [47]. And in our study, according to the Gene Ontology classification of the differentially expressed genes after silencing *LmFTZ-F1*s, cellular process was the largest subcategory in the biological process category, which indicated that the genes involved in cell proliferation underwent dramatic changes after silencing *LmFTZ-F1*s, resulting in a disrupted epidermal cell arrangement. In *Caenorhabditis elegans*, the nuclear receptor gene nhr-25, an *FTZ-F1* homologous gene, is involved in the regulation of epidermal cell development, and the disruption of cell–cell junctions and/or fusion can be caused by mutations in the nhr-25 gene [48], which provided a more robust foundation for silencing *LmFTZ-F1*s in modulating the arrangement of epidermal cells. In addition, hexamerin protein expression was significantly down-regulated following the knockdown of *LmFTZ-F1*s. Hexamerin proteins are widespread in insects, accumulate at extraordinarily high concentrations in the nymphal stages, and have been reported to be involved in cuticle formation in insects [49]. For example, the silencing of the hexamerin protein *Hex-2* gene in combination with JH treatment can cause significant deleterious effects on termite cuticle formation and molting [50]. And some genes encoding chitin synthesis enzymes (trehalase and glutamine-fructose-6-phosphate transaminase), transcription factors (BTB/POZ domain-containing protein 9), and cytochrome P450 enzymes (*CYP303A1* and *CYP4C1*) were significantly down-regulated in the ds*LmFTZ-F1*s-injected group compared to those in the control (Table S3, Figure 6D,E). Silencing these genes blocks molting and leads to insect death; for example, in *T. castaneum*, the silencing of the trehalase gene led to molting deformities by regulating chitin synthesis [51]. The injection of trehazolin, a trehalase inhibitor, can induce death in *L. migratoria* [52]. In *L. migratoria*, the silencing of the BTB domain-containing protein 6 gene affected the transition from nymph to adult [53]. The knockdown of *LmCYP303A1* disturbed nymph–adult molting, leading to death in *L. migratoria* [54]. Therefore, the down-regulation of these apoptotic genes subsequent to the co-silencing of *LmFTZ-F1-X1* and *LmFTZ-F1-X2* may underlie the pre-molt mortality observed in *L. migratoria*. In summary, *LmFTZ-F1* is involved in nymph–nymph ecdysis, possibly by regulating the expression of related genes involved in cuticle formation, chitin synthesis, and other key molting processes; however, further work is needed to clarify the specific regulatory mechanism between *LmFTZ-F1* and these molting genes.

## 5. Conclusions

In the present study, two isoforms of *LmFTZ-F1* (*LmFTZ-F1-X1* and *LmFTZ-F1-X2*) were identified in *L. migratoria*. Their protein sequences contained the conserved domains of the nuclear receptor FTZ-F1 and were distinguished based on the variable N-terminal domain. The expression profiles showed that they were highly expressed in the integument prior to molting. The separate silencing of *LmFTZ-F1-X1* and *LmFTZ-F1-X2* did not affect the normal development of third-instar nymphs, but the simultaneous knockdown of *LmFTZ-F1-X1* and *LmFTZ-F1-X2* caused the nymphs to be arrested in the third-instar stage and finally die. The new cuticles were thicker in the ds*LmFTZ-F1*s-injected group than in the control group. Furthermore, RNA-seq and RT-qPCR results after the silencing of *LmFTZ-F1*s showed that some key molting genes encoding cuticle proteins, chitin synthesis enzymes, hexamerin-like proteins, transcription factors, cytochrome P450 enzymes, and nuclear receptors were significantly differentially expressed between the ds*GFP*- and ds*LmFTZ-F1*s-injected groups. Taken together, *LmFTZ-F1* is involved in nymph–nymph molting by regulating genes involved in cuticle formation, chitin synthesis, and other key molting processes in *L. migratoria*.

## Data Availability

The data that support the findings of this study are available on reasonable request from the first and corresponding author.

## Supplementary Materials

The following supporting information can be downloaded at: https://www.mdpi.com/article/10.3390/insects15040237/s1, Figure S1: Gene expression analysis on day 5 of the third instar nymphs injected with ds*LmFTZ-F1-X1* or ds*LmFTZ-F1-X2*; Table S1: Accession numbers of insect FTZ-Fls; Table S2: Primers used in experiments; Table S3: The annotation of differentially expressed genes between ds*GFP*- and ds*LmFTZ-F1*s-injected groups.
