# Supplementary material for: Nuclear Receptor FTZ-F1 Controls Locust Molt by Regulating the Molting Process of Locusta migratoria
Source: Insects. 2024 Mar 29;15(4):237. doi: 10.3390/insects15040237 (PMC11050008; doi:10.3390/insects15040237)
Supplement: Supplementary file 1 [file insects-15-00237-s001.zip › insects-2913605-supplementary.pdf]

Table S1. Accession numbers of insect FTZ-Fls

Order	Species (gene name)	Accession numbers
**Thysanoptera**	*Frankliniella occidentalis* (FTZ-F1-X1)	XP_052123279.1
	Frankliniella occidentalis (FTZ-F1-X2)	XP_052123280.1
	Thrips palmi (FTZ-F1-X1)	XP_034233825.1
	Thrips palmi (FTZ-F1-X2)	XP_034233826.1
**Hemiptera**	*Myzus persicae* (FTZ-F1)	XP_022165907.1
	Diaphorina citri (FTZ-F1)	XP_026684343.1
	Acyrthosiphon pisum (FTZ-F1)	XP_029344120.1
	Melanaphis sacchari (FTZ-F1)	XP_025196573.1
	Rhopalosiphum maidis (FTZ-F1)	XP_026804562.1
	Nilaparvata lugens (FTZ-F1-X1)	APA21469.1
	Nilaparvata lugens (FTZ-F1-X2)	APU50621.1
**Lepidoptera**	*Aricia agestis* (FTZ-F1-X1)	XP_041986656.1
	Aricia agestis (FTZ-F1-X2)	XP_041986657.1
	Aricia agestis (FTZ-F1-X3)	XP_041986658.1
	Bombyx mori (FTZ-F1)	NP_001037528.2
	Eumeta japonica (FTZ-F1)	GBP13185.1
	Plutella xylostella (FTZ-F1)	KAG7313439.1
**Diptera**	*Drosophila melanogaster* (FTZ-F1-X1)	AAN11667.1
	Drosophila melanogaster (FTZ-F1-X2)	NP_524143.2
	Drosophila busckii (FTZ-F1)	ALC45036.1
	Zeugodacus cucurbitae (FTZ-F1)	JAD13561.1
	Lucilia cuprina (FTZ-F1)	KAI8117233.1
	Aedes aegypti (FTZ-F1)	AAF82307.1
**Hymenoptera**	*Apis mellifera* (FTZ-F1)	XP_006557455.1
	Apis cerana (FTZ-F1)	XP_016904299.1
	Bombus huntii (FTZ-F1)	XP_050469880.1
	Bombus affinis (FTZ-F1)	XP_050588532.1
	Osmia lignaria (FTZ-F1)	XP_034172432.1
	Colletes gigas (FTZ-F1-X1)	XP_043249000.1
	Colletes gigas (FTZ-F1-X2)	XP_043249002.1
	Cotesia glomerata (FTZ-F1-X2)	XP_044587614.1
	Venturia canescens (FTZ-F1-X1)	XP_043285896.1
	Venturia canescens (FTZ-F1-X2)	XP_043285897.1
	Venturia canescens (FTZ-F1-X3)	XP_043285898.1
**Coleoptera**	*Sitophilus oryzae* (FTZ-F1)	XP_030759901.1
	Lasioderma serricorne (FTZ-F1)	ULO04606.1
	Dermestes maculatus (FTZ-F1)	ATU89126.1
	Zophobas morio (FTZ-F1)	KAJ3662564.1

	Tribolium castaneum (FTZ-F1)	KYB28389.1
	Tenebrio molitor (FTZ-F1)	CAH1366627.1
	Phyllotreta striolata (FTZ-F1)	CAH1187269.1
	Leptinotarsa decemlineata (FTZ-F1-X1)	AJF93908.1
	Leptinotarsa decemlineata (FTZ-F1-X2)	AJF93909.1
**Odonata**	*Ischnura elegans* (FTZ-F1-X1)	XP_046390746.1
	Ischnura elegans (FTZ-F1-X2)	XP_046390747.1
**Isoptera**	*Cryptotermes secundus* (FTZ-F1-X1)	XP_023711376.1
	Cryptotermes secundus (FTZ-F1-X2)	XP_023711377.1
	Cryptotermes secundus (FTZ-F1-X3)	XP_023711378.1
**Blattaria**	*Blattella germanica* (FTZ-F1)	CAQ57670.1
**Orthoptera**	*Schistocerca gregaria* (FTZ-F1)	XP_049828864.1
	Schistocerca piceifrons (FTZ-F1)	XP_047098390.1
	Schistocerca nitens (FTZ-F1-X1)	XP_049810469.1
	Schistocerca nitens (FTZ-F1-X2)	XP_049810477.1
	Schistocerca cancellata (FTZ-F1)	XP_049784431.1
	Schistocerca americana (FTZ-F1)	XP_046996266.1

Table S2. Primers used in experiments

Primer name	Sequence (5'-3')	Application
LmFTZ-F1-RNAiF	TAATACGACTCACTATAGGGTCGACTGGGCGCGAAACTCCG	RNAi for two *LmFTZ-F1*s depletion
LmFTZ-F1-RNAiR	TAATACGACTCACTATAGGGCTATTTACGCTTTGCGTGCAA	RNAi for two *LmFTZ-F1*s depletion
LmFTZ-F1-X1-RNAiF	TAATACGACTCACTATAGGGATGCTATTGGACATGGAGCA	RNAi for *LmFTZ-F1X1* depletion
LmFTZ-F1-X1-RNAiR	TAATACGACTCACTATAGGGACGAGGCGTCCATGTTACC	RNAi for *LmFTZ-F1X1* depletion
LmFTZ-F1-X2-RNAiF	TAATACGACTCACTATAGGGATGCATGAGGAGACTGCTAC	RNAi for *LmFTZ-F1X2* depletion
LmFTZ-F1-X2-RNAiR	TAATACGACTCACTATAGGGCTTGTAGTAAGCCCGATGAG	RNAi for *LmFTZ-F1X2* depletion
LmFTZ-F1-X1-qF	CATTCCGCCGCTATGGTCTA	qPCR for expression profile analysis
LmFTZ-F1-X1-qR	GCCACGCTGGTCGTCAC	qPCR for expression profile analysis
LmFTZ-F1-X1-91-qF	GTGTCGTACAGACATCCTCCC	qPCR for RNAi experiments
LmFTZ-F1-X1-91-qR	ACCTTGGATACTCGCTTCCC	qPCR for RNAi experiments
LmFTZ-F1-X2-qF	GCTATCGACTATGCGGGTGG	qPCR for expression profile analysis
LmFTZ-F1-X2-qR	TCGCACGCAGAATCTTCCTA	qPCR for expression profile analysis
LmFTZ-F1-X2-121- qF	AGCTCAAGAACAAGACAGCGT	qPCR for RNAi experiments
LmFTZ-F1-X2-121- qR	GGACTGTGTAGTCGTAGCGG	qPCR for RNAi experiments
LmEF-1α-qF	AACATCGTCGTCATTGGTCA	qPCR for RNAi experiments
LmEF-1α-qR	GTTCAGCCTTCAGCTTGTCC	qPCR for RNAi experiments
LmGAPDH-qF	AGGCCAAGGTCAAGGAAGCT	qPCR for RNAi experiments
LmGAPDH-qR	GAATGGCAGTCACCAATGAAGTC	qPCR for RNAi experiments
LOCMI13131-qF	TCCCCTACACCTACCAGAGC	qPCR for transcriptome verification
LOCMI13131-qR	AGTAGATGGGGTAGCCGGTG	qPCR for transcriptome verification
LOCMI13137-qF	GAGACCAGGAAAGCAGTGCC	qPCR for transcriptome verification
LOCMI13137-qR	AGGATGACAACGGCGAACAT	qPCR for transcriptome verification
NewGene_9906-qF	CACGTCGAAATAAGGTGGCG	qPCR for transcriptome verification
NewGene_9906-qR	CTGACCCAGGCGCATTTTTAT	qPCR for transcriptome verification
LOCMI04988-qF	TGGCCTACTCCGGACTCTAC	qPCR for transcriptome verification
LOCMI04988-qR	GCTCAGTAGTGGTAGGCGAC	qPCR for transcriptome verification
LOCMI16712-qF	CGACAAGCTGGTGACGTACT	qPCR for transcriptome verification
LOCMI16712-qR	TAGTTGAAGGGCTTGTGCGT	qPCR for transcriptome verification
LOCMI15494-qF	TACTACGGCATCAAGGCGAC	qPCR for transcriptome verification
LOCMI15494-qR	TGCCAAGGTACTGGACGAAC	qPCR for transcriptome verification
LOCMI09561-qF	GGCGTGATTCCGAGAGACAT	qPCR for transcriptome verification
LOCMI09561-qR	GATAATGTCCGTGGCGTCCT	qPCR for transcriptome verification
LOCMI08410-qF	CGTTCGTGGACATGATGCAG	qPCR for transcriptome verification
LOCMI08410-qR	GCCTGCTGGAAGTTGTCGT	qPCR for transcriptome verification
LOCMI07002-qF	GGCCGAGGTTACAACTTTGC	qPCR for transcriptome verification
LOCMI07002-qR	CCAGAGCAAGTGGTCCATGT	qPCR for transcriptome verification
LOCMI17468-qF	CGACTCCAGCCAAGAGTGTT	qPCR for transcriptome verification
LOCMI17468-qR	TGAGTGGATGCACGGTACAC	qPCR for transcriptome verification

NewGene_5955-qF	ACTGCTTCTCATCATTGCAGC	qPCR for transcriptome verification
NewGene_5955-qR	AGCTGCCAGTAAGCCTCTTG	qPCR for transcriptome verification
LOCMI16180-qF	TGCTGGCTGGGATTCGATAC	qPCR for transcriptome verification
LOCMI16180-qR	AGGCAACCCACCATGTCAAT	qPCR for transcriptome verification
LOCMI17475-qF	GGCTTCGTCAGCGACTACAT	qPCR for transcriptome verification
LOCMI17475-qR	TGTGCCAGTTTCGCTTGTTC	qPCR for transcriptome verification
LOCMI16491-qF	GAAGGTGGAGGACGAGGTG	qPCR for transcriptome verification
LOCMI16491-qR	TGCCGTTGTAGGCGGACTG	qPCR for transcriptome verification
LOCMI17305-qF	GGGCCGTACAATAACAATGG	qPCR for transcriptome verification
LOCMI17305-qR	GCCACCTCGACCTTGATTTA	qPCR for transcriptome verification

Table S3 The annotation of differentially expressed genes between ds*GFP*- and ds*LmFTZ-F1*s-injected groups

Gene ID	FDR	Log_2FC	Trend	NR_annotation
LOCMI00230	0.03	-1.56	down	translocon-associated protein subunit delta-like (TRAP-delta) [*Coptotermes formosanus*]
LOCMI00320	0.02	-1.42	down	nucleoside diphosphate kinase [*Melanoplus sanguinipes*]
LOCMI00395	0.04	-2.13	down	membrane metallo-endopeptidase-like 1 protein, partial [*Melanoplus sanguinipes*]
LOCMI00502	0.00	-0.93	down	40S ribosomal protein S21 [*Sitophilus oryzae*]
LOCMI00781	0.00	-4.17	down	uncharacterized protein LOC108632050 isoform X1 [*Ceratina calcarata*]
LOCMI00915	0.04	-1.09	down	signal peptidase complex subunit 1 [*Cryptotermes secundus*]
LOCMI01052	0.00	-4.05	down	potassium channel subfamily K member 1-like protein, partial [*Neoconocephalus triops*]
LOCMI01056	0.02	-3.85	down	vesicular glutamate transporter 3-like [*Schistocerca cancellata*]
LOCMI01251	0.05	-2.56	down	alpha-tocopherol transfer protein-like [*Anoplophora glabripennis*]
LOCMI01491	0.01	-5.84	down	hypothetical protein Cfor_08098 [*Coptotermes formosanus*]
LOCMI02143	0.03	-2.40	down	hypothetical protein X975_19579, partial [*Stegodyphus mimosarum*]
LOCMI02197	0.04	-2.95	down	clavesin-2-like [*Zootermopsis nevadensis*]
LOCMI02726	0.00	-3.91	down	hypothetical protein B566_EDAN012018 [*Ephemera danica*]
LOCMI02900	0.00	-7.93	down	synaptic vesicle glycoprotein 2B isoform X1 [*Zootermopsis nevadensis*]
LOCMI02930	0.04	-4.02	down	hypothetical protein Cfor_12581, partial [*Coptotermes formosanus*]
LOCMI03351	0.00	-6.78	down	uncharacterized protein LOC105685706 [*Athalia rosae*]
LOCMI03479	0.05	-2.92	down	formimidoyltransferase-cyclodeaminase-like, partial [*Limulus polyphemus*]
LOCMI03688	0.01	-1.99	down	tyrosine-protein phosphatase non-receptor type 13-like [*Thrips palmi*]
LOCMI04441	0.04	-1.03	down	uncharacterized protein LOC110840115 isoform X2 [*Zootermopsis nevadensis*]
LOCMI04909	0.02	-3.48	down	immulectin 1 [*Locusta migratoria*]
LOCMI04988	0.04	-3.19	down	RecName: Full=Cuticle protein 6.4; AltName: Full=LmNCP6.4 [*Locusta migratoria*]
LOCMI05288	0.00	-3.14	down	15-hydroxyprostaglandin dehydrogenase [NAD(+)] [*Cryptotermes secundus*]

LOCMI05366	0.00	-7.09	down	RWD domain-containing protein 4A [*Zootermopsis nevadensis*]
LOCMI06000	0.00	-8.31	down	--
LOCMI06013	0.00	-5.41	down	glycine N-methyltransferase [*Athalia rosae*]
LOCMI06322	0.04	-0.67	down	60S ribosomal protein L37a [*Ctenocephalides felis*]
LOCMI06512	0.00	-7.81	down	Putative defense protein 3 [*Zootermopsis nevadensis*]
LOCMI06602	0.02	-2.96	down	60S ribosomal protein L9 [*Sogatella furcifera*]
LOCMI07002	0.03	-1.75	down	glutamine--fructose-6-phosphate aminotransferase [isomerizing] 2-like isoform X1 [*Zootermopsis nevadensis*]
LOCMI07265	0.03	-1.02	down	putative signal peptidase complex subunit 2 [*Blattella germanica*]
LOCMI07550	0.01	-2.06	down	phosphoacetylglucosamine mutase [*Locusta migratoria manilensis*]
LOCMI07701	0.00	-3.88	down	cuticular protein [*Nilaparvata lugens*]
LOCMI07703	0.00	-3.67	down	endonuclease-reverse transcriptase [*Eyprepocnemis plorans plorans*]
LOCMI07885	0.04	-1.62	down	hypothetical protein Cfor_05696 [*Coptotermes formosanus*]
LOCMI08410	0.01	-4.35	down	trehalase [*Locusta migratoria*]
LOCMI08553	0.04	-0.77	down	PREDICTED: high affinity copper uptake protein 1 isoform X1 [*Fopius arisanus*]
LOCMI08747	0.01	-3.09	down	RecName: Full=Putative defense protein; AltName: Full=Basic 19 kDa hemolymph protein; Flags: Precursor [*Locusta migratoria*]
LOCMI09100	0.00	-1.27	down	hypothetical protein B7P43_G04116 [*Cryptotermes secundus*]
LOCMI09561	0.03	-3.84	down	hexamerin-like protein 2, partial [*Pielomastax soochowensis*]
LOCMI09883	0.02	-2.57	down	lysosomal alpha-mannosidase-like isoform X1 [*Agrilus planipennis*]
LOCMI09998	0.03	-4.74	down	haemolymph juvenile hormone binding [*Cinara cedri*]
LOCMI10344	0.01	-2.79	down	FK506-binding protein 2 [*Cryptotermes secundus*]
LOCMI10487	0.05	-1.34	down	G2/mitotic-specific cyclin-B1-like [*Megalopta genalis*]
LOCMI10612	0.00	-1.38	down	RecName: Full=Protein transport protein Sec61 subunit gamma [*Gryllotalpa orientalis*]
LOCMI10868	0.00	-4.46	down	RecName: Full=Putative defense protein; AltName: Full=Basic 19 kDa hemolymph protein; Flags: Precursor [*Locusta migratoria*]
LOCMI11369	0.02	-3.82	down	PREDICTED: nose resistant to fluoxetine protein 6-like [*Neodiprion lecontei*]
LOCMI11745	0.02	-1.26	down	probable cysteine--tRNA ligase, mitochondrial isoform X1 [*Zootermopsis nevadensis*]
LOCMI11963	0.03	-3.39	down	TPA_exp: hexamerin-like protein 6 [*Locusta migratoria*]

LOCMI12032	0.03	-0.78	down	28S ribosomal protein S24, mitochondrial [*Zootermopsis nevadensis*]
LOCMI12258	0.03	-1.29	down	hypothetical protein GE061_001115 [*Apolygus lucorum*]
LOCMI12679	0.03	-5.72	down	hypothetical protein C0J52_16170 [*Blattella germanica*]
LOCMI12762	0.04	-2.92	down	uncharacterized protein LOC106677882 [*Halyomorpha halys*]
LOCMI13131	0.02	-7.84	down	nymph cuticular protein NCP62 [*Locusta migratoria*]
LOCMI13132	0.01	-6.84	down	nymph cuticular protein NCP9.5 [*Locusta migratoria*]
LOCMI13137	0.01	-6.56	down	nymph cuticular protein NCP9.5 [*Locusta migratoria*]
LOCMI13553	0.01	-1.32	down	translocon-associated protein subunit beta [*Cryptotermes secundus*]
LOCMI13592	0.00	-5.46	down	hypothetical protein C0J52_11186 [*Blattella germanica*]
LOCMI13597	0.02	-0.85	down	PREDICTED: 40S ribosomal protein S2 [*Tribolium castaneum*]
LOCMI14004	0.03	-4.03	down	PREDICTED: serine--pyruvate aminotransferase, mitochondrial [*Nicrophorus vespilloides*]
LOCMI14014	0.00	-3.01	down	yellow protein of the takeout family [*Schistocerca gregaria*]
LOCMI14015	0.00	-3.46	down	RecName: Full=Putative beta-carotene-binding protein [*Schistocerca gregaria*]
LOCMI14358	0.04	-2.78	down	hypothetical protein C0J52_13145 [*Blattella germanica*]
LOCMI14451	0.00	-1.84	down	hypothetical protein C0J52_05160 [*Blattella germanica*]
LOCMI14629	0.01	-0.90	down	glutamine amidotransferase-like class 1 domain-containing protein 3A, mitochondrial isoform X1 [*Cryptotermes secundus*]
LOCMI14640	0.01	-1.04	down	protein SEC13 homolog [*Zootermopsis nevadensis*]
LOCMI15494	0.00	-5.16	down	hexamerin-like protein 1 [*Locusta migratoria*]
LOCMI15658	0.01	-3.53	down	hypothetical protein [*Locusta migratoria*]
LOCMI15659	0.01	-3.42	down	hypothetical protein [*Locusta migratoria*]
LOCMI15699	0.03	-3.50	down	glutathione S-transferase sigma 4 [*Locusta migratoria*]
LOCMI15974	0.02	-1.00	down	1,5-anhydro-D-fructose reductase-like [*Zootermopsis nevadensis*]
LOCMI16163	0.01	-1.70	down	cytochrome P450 6k1-like isoform X1 [*Schistocerca cancellata*]
LOCMI16180	0.00	-3.90	down	probable cytochrome P450 303a1 [*Schistocerca piceifrons*]
LOCMI16252	0.02	-2.08	down	peroxiredoxin-5, mitochondrial isoform X1 [*Zootermopsis nevadensis*]
LOCMI16296	0.00	-2.39	down	Dehydrogenase/reductase SDR family member 11 [*Blattella germanica*]

LOCMI16712	0.01	-5.23	down	hexamerin-like protein 1 [*Locusta migratoria*]
LOCMI17005	0.01	-4.20	down	UDP-glucuronosyltransferase 1-2 [*Locusta migratoria migratoria*]
LOCMI17043	0.04	-2.47	down	hypothetical protein Cfor_00643 [*Coptotermes formosanus*]
LOCMI17468	0.01	-3.93	down	BTB/POZ domain-containing protein 9 [*Thrips palmi*]
LOCMI17475	0.00	-2.65	down	cytochrome P450 4C1-like [*Schistocerca nitens*]
NewGene_11021	0.02	-5.42	down	PREDICTED: RNA-directed DNA polymerase from mobile element jockey-like [*Acropora digitifera*]
NewGene_12561	0.00	-1.70	down	PREDICTED: 60S ribosomal protein L27a [*Neodiprion lecontei*]
NewGene_3448	0.00	-4.71	down	NADH dehydrogenase [ubiquinone] 1 alpha subcomplex subunit 9, mitochondrial [*Cryptotermes secundus*]
NewGene_6583	0.01	-2.45	down	--
NewGene_6825	0.03	-5.28	down	uncharacterized protein LOC116347257 [*Contarinia nasturtii*]
NewGene_8995	0.00	-1.72	down	hypothetical protein B7P43_G02383 [*Cryptotermes secundus*]
NewGene_9906	0.00	-6.42	down	cuticle protein 16.5-like [*Thrips palmi*]
LOCMI01462	0.01	2.00	up	hypothetical protein C0J52_20255 [*Blattella germanica*]
LOCMI01671	0.01	1.63	up	--
LOCMI01748	0.03	1.70	up	hypothetical protein C0J52_19749 [*Blattella germanica*]
LOCMI02075	0.04	3.09	up	putative carbonic anhydrase 3 [*Ctenocephalides felis*]
LOCMI02406	0.01	3.33	up	agrin [*Orussus abietinus*]
LOCMI02506	0.00	2.40	up	protein unc-13 homolog 4B isoform X2 [*Zootermopsis nevadensis*]
LOCMI02673	0.02	1.15	up	hypothetical protein Cfor_12196 [*Coptotermes formosanus*]
LOCMI03339	0.00	1.17	up	inter-alpha-trypsin inhibitor heavy chain H4 isoform X2 [*Cryptotermes secundus*]
LOCMI03392	0.00	2.20	up	xaa-Pro aminopeptidase ApepP [*Cryptotermes secundus*]
LOCMI03517	0.01	1.32	up	uncharacterized protein LOC111861285 isoform X2 [*Cryptotermes secundus*]
LOCMI05259	0.02	2.22	up	hypothetical protein B7P43_G16712 [*Cryptotermes secundus*]
LOCMI05525	0.00	1.09	up	variable lymphocyte receptor [*Locusta migratoria*]
LOCMI06129	0.01	1.27	up	hypothetical protein B7P43_G18203 [*Cryptotermes secundus*]
LOCMI06171	0.00	1.47	up	GATOR complex protein NPRL2-like isoform X1 [*Zootermopsis nevadensis*]

LOCMI06254	0.00	3.14	up	solute carrier family 13 member 5 isoform X1 [*Ischnura elegans*]
LOCMI07087	0.00	1.78	up	synaptojanin-1 isoform X1 [*Cryptotermes secundus*]
LOCMI07559	0.04	1.36	up	brummer [*Locusta migratoria*]
LOCMI07657	0.03	1.91	up	hypothetical protein Cfor_09764, partial [*Coptotermes formosanus*]
LOCMI08798	0.03	1.31	up	PREDICTED: alanine--glyoxylate aminotransferase 2, mitochondrial [*Bemisia tabaci*]
LOCMI08995	0.01	1.31	up	glucose-6-phosphate 1-dehydrogenase isoform X1 [*Zootermopsis nevadensis*]
LOCMI09120	0.02	0.75	up	non-specific lipid-transfer protein [*Thrips palmi*]
LOCMI09618	0.04	1.31	up	phosphatidylinositol phosphatase PTPRQ-like isoform X3 [*Zootermopsis nevadensis*]
LOCMI09721	0.00	9.76	up	hypothetical protein Cfor_12661 [*Coptotermes formosanus*]
LOCMI09827	0.04	1.25	up	glycosylated lysosomal membrane protein A isoform X1 [*Cryptotermes secundus*]
LOCMI10800	0.01	1.91	up	hypothetical protein C0J52_13397 [*Blattella germanica*]
LOCMI11213	0.05	2.49	up	Solute carrier family 22 member 21 [*Cryptotermes secundus*]
LOCMI12862	0.03	1.07	up	glycogen debranching enzyme isoform X1 [*Cryptotermes secundus*]
LOCMI12950	0.05	2.26	up	hypothetical protein C0J52_18891 [*Blattella germanica*]
LOCMI13037	0.02	0.90	up	extended synaptotagmin-2 isoform X2 [*Zootermopsis nevadensis*]
LOCMI13444	0.02	1.42	up	inositol oxygenase [*Nilaparvata lugens*]
LOCMI13445	0.04	3.11	up	inositol oxygenase isoform X1 [*Athalia rosae*]
LOCMI13871	0.03	2.06	up	ornithine decarboxylase [*Cryptotermes secundus*]
LOCMI13876	0.01	1.16	up	DNA-binding protein D-ETS-4 isoform X2 [*Cryptotermes secundus*]
LOCMI14504	0.01	2.10	up	chloride channel protein 2 isoform X1 [*Cryptotermes secundus*]
LOCMI14638	0.04	1.38	up	taiman transcript variant B [*Locusta migratoria*]
LOCMI14658	0.04	1.19	up	hypothetical protein C0J52_15705 [*Blattella germanica*]
LOCMI14840	0.02	0.93	up	hypothetical protein C0J52_15396 [*Blattella germanica*]
LOCMI15262	0.01	0.86	up	EH domain-containing protein 3-like [*Zootermopsis nevadensis*]
LOCMI17560	0.01	1.83	up	RecName: Full=Fatty acid-binding protein, muscle; AltName: Full=M-FABP [*Locusta migratoria*]
NewGene_12730	0.00	3.22	up	solute carrier family 22 member 24-like isoform X3 [*Zootermopsis*

				nevadensis]
NewGene_3599	0.05	1.04	up	hypothetical protein B7P43_G16846, partial [*Cryptotermes secundus*]
NewGene_5180	0.03	1.69	up	cytochrome P450 4c3-like [*Schistocerca nitens*]
NewGene_584	0.00	4.41	up	pro-resilin-like [*Schistocerca piceifrons*]
NewGene_5955	0.01	2.15	up	metabotropic glutamate receptor 3 [*Nephila pilipes*]
NewGene_8598	0.01	1.77	up	alpha-aminoadipic semialdehyde mitochondrial [*Lasius niger*]

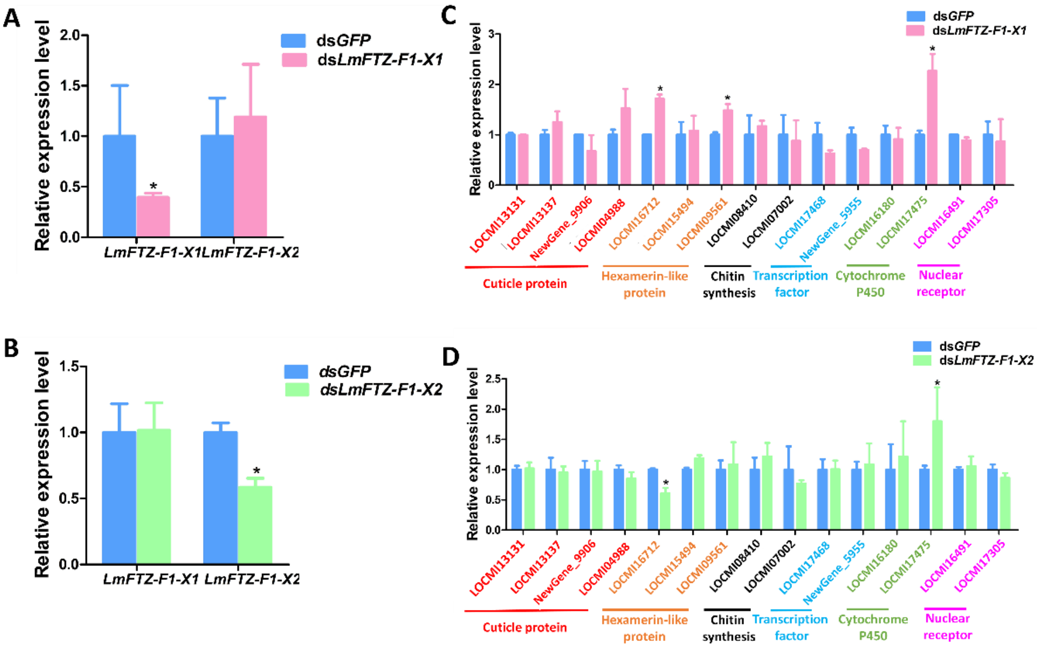

Figure S1. Gene expression analysis on day 5 of the third instar injected with ds*LmFTZ-F1-X1* or ds*LmFTZ-F1-X2*. A, B. Expression analysis of *LmFTZ-F1-X1* and *LmFTZ-F1-X2* on day 5 of the third instar injected with ds*GFP*, ds*LmFTZ-F1-X1* and ds*LmFTZ-F1-X2*. C, D. Expression analysis of DEGs induced by co-silencing of *LmFTZ-F1-X1* and *LmFTZ-F1-X2* on day 5 of the third instar injected with ds*GFP*, ds*LmFTZ-F1-X1* and ds*LmFTZ-F1-X2*. The data was analyzed using the independent sample T-test. The asterisks indicated that there were significant differences between the control and treatment groups (*, $p<0.05$).
